# Omega-3 supplement alters water consumption and plasma fatty acid profile of beef heifers

**DOI:** 10.1093/jas/skaf312

**Published:** 2025-09-09

**Authors:** Kendal L Green, Madison R Kovarna, Ethan R Schlegel, Cody L Wright, Ana C B Menezes, Zachary K F Smith, Jessica N Drum

**Affiliations:** Department of Animal Science, South Dakota State University, Brookings, SD 57007; Department of Animal Science, South Dakota State University, Brookings, SD 57007; New Generation Supplements, Belle Fourche, SD 57717; Department of Animal Science, South Dakota State University, Brookings, SD 57007; Department of Animal Science, South Dakota State University, Brookings, SD 57007; Department of Animal Science, South Dakota State University, Brookings, SD 57007; Department of Animal Science, South Dakota State University, Brookings, SD 57007

**Keywords:** lipids, flaxseed oil, polyunsaturated fatty acid, progesterone, puberty

## Abstract

Flaxseed oil contains elevated levels of omega-3 fatty acids (n-3 FAs), which have been shown to impact reproductive performance. This study aimed to determine the effects of a flaxseed oil-based supplement (FLAX) on reproductive parameters, feeding behavior, and lipid profile in beef heifers. Sixty Angus and Simmental × Angus heifers (14 months old ± 2 months), blocked by full body weight (396.79 ± 33.78 kg) ± SD and antral follicle count, were randomly assigned to one of two ad libitum supplementation treatments: a commercial loose mineral supplement (CON; *n *= 30) or a FLAX (*n* = 30) for 8 wk. Heifers were individually fed by an automated feeding system with a basal diet (total mixed ration, TMR), a mix of corn silage, grass hay, and dried distillers grains plus solubles (DDGS) offered ad libitum. Water and supplements were provided in separate feeders to measure intake and were compiled over 24 h for each animal by the Insentec system. In week 5, heifers were enrolled in a fixed-time artificial insemination (FTAI) protocol. The presence of a corpus luteum (CL) was recorded weekly, as well as the largest follicle diameter and CL presence on days of the FTAI protocol (D0, D7, and D9). Puberty attainment was determined when a CL could be visualized by ultrasound and/or the blood sample contained concentrations of P4 above the threshold level of 1 ng/mL. Pregnancy diagnosis [P/artificial insemination (AI); % (*n*)] was performed at ∼30 d and ∼60 d following AI. Plasma samples were collected once weekly and on days 0, 7, and 9 of the FTAI protocol for circulating progesterone (P4), estradiol (E2), and fatty acid (FA) profiles. Water intake in kilograms per day (kg/d) was lower in FLAX, while TMR intake (kg/d) tended (*P *= 0.08) to be greater in FLAX compared to CON. Additionally, puberty tended to be attained faster (*P *= 0.10), and P4 on D9 tended to be lower (*P *= 0.09) for FLAX heifers. Concentrations of α-linolenic and linoleic acids were greater in FLAX (*P *< 0.03). In addition, eicosapentaenoic, arachidonic, and total polyunsaturated fatty acids tended to be greater for heifers in the FLAX group (*P *> 0.07). In conclusion, supplementation of n-3 FAs caused lower water intake, higher plasma FA, and a tendency for faster puberty attainment. In conjunction, these findings offer data to support the use of a flaxseed oil-based supplement as a positive supplementation strategy for beef heifers to induce an optimal endocrine environment and potentially anticipate puberty.

## Teaser

Flaxseed oil is rich in omega-3 fatty acids, which have been found to increase fertility and improve reproductive function in cattle. Flaxseed oil-based supplementation, feeding behavior, and circulating lipid profile in beef heifers offering a practical strategy to improve heifer production.

## Introduction

Longevity and lifetime productivity are two factors that greatly impact profitability for a cow–calf producer (Perry and Cushman, 2013). Heifers conceiving earlier in the breeding season calve earlier and wean heavier calves (Funston et al., 2012; Cushman et al., 2013). Further, conception during the first 21 d of the breeding season has been linked to pubertal status (Cushman et al., 2013). Nutritional management has become a vital factor in accelerating the onset of puberty. It optimizes reproductive success over a cow’s lifetime by directly connecting puberty attainment to longevity and productivity (Patterson et al., 1992; Perry and Cushman, 2013; Wathes et al., 2014).

Polyunsaturated fatty acid (PUFA) supplementation has gained popularity over the last several decades, aiming to improve performance. Essential FAs, such as linoleic (LA) and α-linolenic (ALA), cannot be synthesized by the animal; therefore, they must be included in the diet (Wathes et al., 2007). These essential fatty acids are necessary for numerous processes, including growth, reproduction, vision, and brain development (Gurr et al., 2016). Multiple studies have shown the positive effects of PUFAs on fertility and cyclicity (Bender et al., 2010; Gulliver et al., 2012; Maranesi et al., 2018). Specific strategies with PUFA supplementation have also shown promising results in advancing the onset of puberty. Lammoglia et al. (2000) observed that beef heifers supplemented with safflower seed tended to reach puberty earlier in the breeding season compared with their counterparts receiving no supplementation.

Even with all these benefits, supplementation programs can still be labor-intensive, increasing production costs in cow–calf systems, which may discourage producers from implementing the techniques (Miller et al., 2001). The use of a molasses-based low-moisture block (LMB) helps to alleviate these concerns, as it offers a self-fed form of supplementation, providing energy, protein, and custom nutrients to forage-fed cattle (Moriel et al., 2019; Pickett et al., 2023). The manufacturing of the LMB uses a continuous flow strategy. This process starts by heating a mixture of molasses and flaxseed oil, high in n-3 PUFA (Gulliver et al., 2012), to evaporate moisture and then incorporate protein, vitamins, and minerals into the desired composition (Miller, 2012; Drouillard, 2013). The result is a dense block that allows resistance to temperature changes and can be fed year-round (Drouillard, 2013). However, little is known about how flaxseed oil-based LMBs alter performance and feeding behavior, as well as how much would effectively be delivered to affect the lipid profile in the circulation.

We hypothesized that supplementing heifers with flaxseed oil presented as an LMB would impact feeding behavior and positively affect reproductive performance in beef heifers, in terms of optimizing P4 [high during follicular growth and low at artificial insemination (AI)] and E2 (high at the time of AI) concentrations, as well as increasing follicle diameter. The overall objective of this study was to determine the effects of a flaxseed oil-based supplement (FLAX) on reproductive parameters, feeding behavior, and lipid profile in beef heifers. Specifically, we aimed to evaluate the supplementation effect on intake, circulating steroid hormones, FAs, fertility, and puberty attainment in beef heifers.

## Materials and Methods

### Animals, experimental design, and dietary treatments

All procedures involving animals were reviewed and approved by the Institutional Animal Care and Use Committee of the South Dakota State University (IACUC protocol approval number: 2302-029A)

Sixty Angus and Simmental × Angus heifers were blocked by full body weight (BW = 396.79 ± 33.78 kg) and antral follicle count, and randomly assigned to one of two supplementation treatments: 1) loose mineral supplement (CON; *n* = 30) or 2) flaxseed oil-based blocks (FLAX; *n* = 30). The FLAX treatment consisted of a commercial LMB (FlaxLic, New Generations, Belle Fourche, SD) composed of beet molasses, ground flaxseed, flaxseed oil, soybean meal, minerals, and vitamins (Table 2). The loose mineral premix provided macro and trace minerals and vitamins A, D, and E to meet or exceed NASEM (2016) requirements (Purina Wind and Rain All Season 7.5 Complete Mineral^®^, Land O’ Lakes, Inc., Arden Hills, MN). Assurance levels for both treatment supplements are detailed in Table 2. The FA profile and composition of the FLAX treatment are shown in Table 3.

**Table 2.** Composition of respective supplements fed to heifers over an 8-wk trial period; company guaranteed analysis

	Assurance Levels^3^			
	FLAX^1^		CON^2^	
Item	Min	Max	Min	Max
Crude protein, g/kg of DM	120.0	–	–	–
Crude fat, g/kg of DM	150.0	–	–	–
Crude fiber, g/kg of DM	–	20.0	–	–
ADF, g/kg of DM	–	25.0	–	–
Minerals				
Ca, g/kg of DM	10.0	15.0	135.0	162.0
P, g/kg of DM	10.0	25.0	75.0	
NaCl, g/kg of DM		–	180.0	216.0
Mg, g/kg of DM		–	10.0	–
K, g/kg of DM	25.0	–	10.0	–
Mn, mg/kg of DM	1,000.0	–	4,800	–
Co, mg/kg of DM	3.0	–	19	–
Cu, mg/kg of DM	300.0	–	1,200	–
I, mg/kg of DM	15.0	–	60	–
Se, mg/kg of DM	6.6	–	27	–
Zn, mg/kg of DM	1,200.0	–	3,600	–
Vitamins, IU/kg of DM				
A	176,000.0	–	661,500	–
D	17,640	–	66,150	–
E	176.4	–	661.5	–
Omega-3 Fatty Acids, g/kg of DM	70.0	–	–	–

1 FlaxLic: free choice low-moisture block (New Generation Supplements., Belle Fourche, SD); ingredients: beet molasses, ground flaxseed, flaxseed oil, soybean meal, monocalcium phosphate, limestone, zinc sulfate, manganese sulfate, copper chloride, sodium selenite, ethylenediamine dihydroiodide, cobalt carbonate, vitamin A, acetate, vitamin D3 supplement, vitamin E supplement.

2 Purina Wind and Rain Storm All Season 7.5 Complete Mineral (Land O’ Lakes, Inc., Arden Hills, MN); ingredients: dicalcium phosphate, monocalcium phosphate, processed grain by-products, plant protein products, calcium carbonate, molasses products, salt, mineral oil, potassium chloride, magnesium oxide, ferric oxide, vitamin E supplement, vitamin A supplement, lignin sulfonate, cobalt carbonate, manganese sulfate, ethylenediamine dihydroiodide, zinc sulfate, copper chloride, vitamin D3 supplement, natural and artificial flavors, and sodium selenite.

3 Data are shown as a comparison of mineral and vitamin profiles between treatment supplements.

Abbreviations: DM, dry matter; g, grams; IU, international units; kg, kilograms; mg, milligrams.

**Table 3.** Fatty acid composition of flaxseed oil-based low moisture block fed to heifers over an 8-wk trial period; company guaranteed analysis

Fatty acid	Level (g/100g)
Capric (C10:0)	<0.01
Lauric (C12:0)	<0.01
Tridecanoic (C13:0)	<0.01
Myristic (C14:0)	0.01
Pentadecanoic (C15:0)	<0.01
Palmitic (C16:0)	0.82
Heptadecanoic (C17:0)	0.012
Stearic (C18:0)	0.60
Oleic (C18:1 Cis)	3.06
Linoleic (C18:2 Cis)	2.40
gamma-Linolenic (C18:3 gamma)	<0.01
Nonadecanoic (C19:0)	<0.01
alpha-Linolenic (C18:3 alpha)	7.37
Arachidic (C20:0)	0.03
Arachidonic (C20:4)	<0.01
Eicosapentaenoic (C20:5)	<0.01
Heneicosanoic (C21:0)	<0.01
Behenic (C22:0)	<0.01
Docosapentaenoic (C22:5)	<0.01
Docosahexaenoic (C22:6)	<0.01
Saturated fat (total)	1.54
Polyunsaturated fats (total)	9.80
Monounsaturated fats (total)	3.11
Trans fatty acids (total)	0.01
Omega 3 fatty acids (total)	7.38
Omega 6 fatty acids (total)	2.41
Omega 9 fatty acids (total)	3.06

Abbreviation: g, grams.

**Figure 1.**
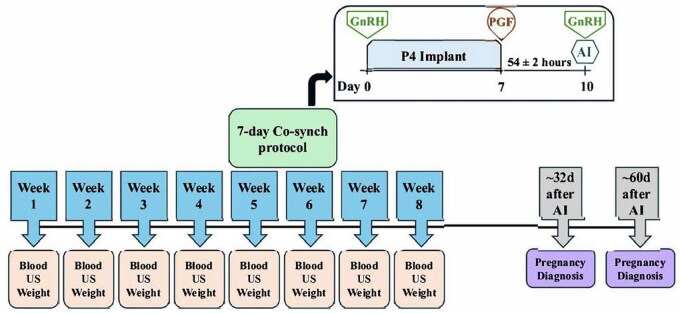
Timeline of sample days occurring once per week including blood sampling, body weight, ovarian ultrasound scans, and synchronization protocol over the 8-wk trial duration. Pregnancy diagnosis by ultrasound was conducted ∼32 d and ∼60 d after AI. Abbreviations: AI, artificial insemination; GnRH, gonadotropin-releasing hormone (100 mcg gonadorelin, Factrel, Zoetis); PGF, prostaglandin F2α (25 mcg dinoprost, Lutalyse HighCon, Zoetis); P4 implant, intravaginal progesterone implant (1.38 g, Eazi-Breed CIDR, InterAg); US, ovarian ultrasound scan.

Heifers were housed at the SDSU Cow–Calf Education and Research Facility in two group pens (*n *= 30 heifers/pen) with equal distribution of treatments per pen (*n* = 15 FLAX and *n* = 15 CON in each pen). Pens were equipped with 12 automated Insentec feeders and 2 automated Insentec waterers per pen (Insentec RIC, Hokofarm, Marknesse, the Netherlands). In each pen, the distribution of diets/supplements in the feeders was as follows: eight feeders with total mixed ration (TMR; consisting of corn silage, grass hay, and DDGS), two feeders with flaxseed oil-based LMBs, and two feeders with loose mineral supplement. The proportion of ingredients and chemical composition of the TMR are outlined in Table 1. Heifers were fitted with a radio-frequency identification tag in the left ear before the beginning of the experiment to allow for the use of the Insentec automated feeding system. This also allowed us to assign heifers to their respective supplement feeders. For instance, in each pen, CON heifers were only allowed to access the two feeders with the loose supplement, while FLAX heifers were only allowed to access the two feeders containing the flaxseed oil-based LMB. Further, all heifers had equal access to the eight feeders containing TMR. The heifers had been housed in those pens previously, so there was no need to adapt the heifers to the feeding system regarding the TMR consumption. All heifers had consumed treatments from the respective supplement feeders after 10 d of the trial period had started. The TMR and supplements were offered ad libitum, and the heifers had free access to water. Heifers received treatments from 6-wk pre-breeding to 2-wk post-breeding. The timeline of sampling events, including blood, ovarian scans, BW, and synchronization protocol, is described in detail below and shown in Figure 1.

**Table 1.** Proportion of ingredients and chemical composition of the basal diet

Item			Quantity
Ingredient composition (% of dry matter)			
Grass hay			61.1
Corn silage			21.0
Dried distiller’s grain plus solubles			17.9
Chemical composition^1^			
Crude protein (%)			12.0
NEm (Mcal/kg)			1.48
NEg (Mcal/kg)			0.88

1 Based on analyzed chemical composition of individual ingredients.

Abbreviations: kg, kilogram, Mcal, megacalorie; NEg, net energy for gain; NEm, net energy for maintenance.

### Feed intake, feeding behavior, and BW

Individual feed intake and feeding behavior characteristics were monitored via the Insentec system (Insentec RIC, Hokofarm, Marknesse, the Netherlands). The system compiles individual intake data over 24 h for each animal. Feeding and water behavior and intake measurements were quantified as time spent eating/drinking in minutes and feed or water intake in kg/d, respectively. Additionally, LMBs and loose minerals were also manually weighed before being placed in the bunks and after they were emptied out to monitor treatment consumption. The weight of the full treatment container was subtracted from the empty container weight to confirm the Insentec measurements.

BW measurements were taken on 2 consecutive days at the beginning of the experiment and on a weekly basis throughout the experiment in the a.m. before feed delivery.

### Ultrasonography and blood collections

Transrectal ultrasonography was performed weekly alongside blood collection and BW measurements using a 7.5-MHz linear probe (Ibex Evo II, E.I. Medical Imaging, Loveland, CO). During each scan, the ovaries were evaluated to record the diameter of the largest follicle (largest follicle diameter, LFD) and the presence of a corpus luteum (CL). Additional blood samples and ovarian scans were collected on days 0, 7, and 9 of the synchronization protocol.

Blood was collected via jugular venipuncture using an 18-gage hypodermic needle attached to a Vacutainer–Leur adapter and evacuated tube. Approximately 10 mL of blood was collected into commercial heparinized tubes (Vacutainer, 10 mL; Becton, Dickinson and Company, Franklin Lakes, NJ). Samples were centrifuged at 2,500*g* for 15 min at 4 °C to separate plasma, which was then stored at −20 °C on the day of collection until analysis.

### Synchronization protocol, AI, and pregnancy diagnosis

Heifers were considered pubertal when a CL could be visualized by ultrasound and/or the blood sample contained concentrations of P4 above the threshold level of 1 ng/mL, indicating luteal activity (Henricks et al., 1971; Funston and Larson, 2011). Heifers that did not attain puberty until week 5 were considered to have been induced to puberty by the synchronization protocol as previously described (Hall et al., 1997).

Heifers were synchronized using a 7-day Co-synch + CIDR protocol (Bridges et al., 2014) starting on week 5 (Figure 1). Briefly, on day 0, heifers received GnRH (gonadotropin-releasing hormone; 100 mcg gonadorelin; Factrel, Zoetis) concomitantly with a P4 implant (1.38 g P4, Eazi-Breed CIDR, InterAg, Hamilton, New Zealand) insertion. Seven days later, they received PGF (prostaglandin F2α; 25 mcg dinoprost; Lutalyse HighCon, Zoetis) concomitant with P4 implant withdrawal. AI was performed 52–56 h after P4 implant removal and concomitant with the administration of another GnRH injection (100 mcg). Pregnancy diagnosis was determined at approximately 32 d and confirmed 60 d after AI by ultrasonography to determine pregnancy per AI (P/AI). A viable embryo/fetus with a heartbeat was considered a positive pregnancy status.

### Progesterone and estradiol concentration

Radioimmunoassay was utilized to determine the concentration of P4 on D0, D7, D9, and E2 concentration on D7 and D9 of the fixed-time artificial insemination (FTAI) protocol, as well as for the weekly samples. A 125 I-radioimmunoassay kit for P4 (Progesterone Coated Tube RIA Kit, MP Biomedicals, Solon, OH) was utilized according to the manufacturer’s instructions and has been validated for use in bovine serum (Skenandore et al., 2017). The sensitivity of the assay was 0.08 ng/mL, and intra- and inter-assay coefficients of variation averaged 8.7% and 12.9%, respectively.

For E2 analysis, the radioimmunoassay protocol adapted from Clapper et al. (2022), with slight modifications, was used. Briefly, estradiol-17β (E8875; Sigma Life Science, St Louis, MO) was used as a standard, and the radioiodinated E2 (#07138226, MP Biomedicals, Solon, OH) was used as a tracer. Antisera (GDN#244 anti-estradiol-17β-6-BSA; Fort Collins, CO) was used at a dilution of 1:425,000 in steroid buffer media. Plasma was extracted with 4 mL of methyl tert-butyl ether. The sensitivity of the assay was 0.11 pg/mL, and intra-assay, and inter-assay coefficients of variation were 9.8% and 14.2%, respectively.

### Plasma lipid profile analysis

The lipid profile analysis was performed on plasma samples from week 1 and week 8 using a two-step procedure of FA methyl ester synthesis and gas chromatography–flame ionization detection, as previously described by O’Fallon et al. (2007). Fatty acids were identified by comparing their retention times with FA methyl standards, as described by O’Fallon et al. (2007).

### Statistical analysis

Data are presented as a completely randomized design with individual heifers as the experimental unit. All data were tested for normality using the UNIVARIATE procedure of SAS (v9.4, SAS Institute Inc, Cary, NC).

Continuous variables such as P4, E2, and LFD were analyzed using the MIXED procedure as repeated measures with fixed effects of treatment, day, and their interactions. The subject was a heifer within treatment. When needed, P4 and FA concentrations were logarithmically transformed for normality. For variables with multiple observations per heifer, such as TMR consumption, average daily gain (ADG), treatment intake, water intake, and FA concentrations, repeated measures with model terms including treatment, week, and their respective interaction were used. To account for individual variation, heifers within week and treatment were included as random effects. Week 6 was omitted from the water intake analysis due to a malfunction that resulted in no data being collected for 3 d of that week. A total of four heifers were removed from all analyses as their intake average from week 2 to week 8 was ≥2 SDs less than the product-recommended intake (0.5 kg/head/d).

Binomial variables, such as P/AI and CL presence by week 5, were analyzed using the GLIMMIX procedure of SAS. The LIFETEST procedure was used to perform a survival analysis for puberty attainment by the start of synchronization.

Covariate structures, including compound symmetry, autoregressive (1), variance components, and unstructured, were tested where appropriate. The structure with the lowest Akaike information criterion and Bayesian information criterion, unstructured, was used for each model. The PDIFF function of SAS was used to separate means with a Tukey–Kramer adjustment, and results are shown as LSM ± SEM (least squares mean ± SEM). Significance was considered at *P *≤ 0.05 for all analyses, and tendencies were considered at *P *> 0.05 and *P *≤ 0.10.

## Results

### Feeding behavior, intake, and performance

While their interaction was not significant (*P *= 0.68), there was a tendency (*P *= 0.08) for TMR intake to be greater for FLAX than CON. Intake of TMR showed a significant treatment × week interaction (*P *= 0.05), varying through the weeks (*P *= 0.003), increasing up to week 4 and then exhibiting a decrease. There was no treatment × week interaction (*P *= 0.32; Table 4) for TMR intake duration, and no difference was exhibited by treatment (*P *= 0.24) or week (*P *= 0.18).

**Table 4.** Supplementation strategy (CON, loose mineral supplement vs. FLAX delivered via low moisture block) impact on performance and intake parameters of beef heifers

	Treatments^1^			*P*-values		
Items^2^	FLAX	CON	SEM^3^	Treatment	Week	Treatment x Week
Initial BW, kg	396.72	394.38	10.70	0.83	-	-
Final BW, kg	425.70	422.85	9.43	0.76	-	-
ADG, kg/d	1.30	1.31	0.19	0.97	0.0001	0.03
Water intake, kg/d	32.06	33.93	0.36	0.04	0.0001	0.05
TMR intake, kg/d	12.90	12.02	0.17	0.08	0.003	0.68
TMR intake duration, min/d	144.08	149.27	5.57	0.24	0.18	0.32
Supplement intake, kg/d	0.72	0.18	0.04	0.0001	0.0001	0.0001
Supplement intake duration, min/d	61.39	0.10	2.71	0.0001	0.0001	0.0001

1 Treatments were applied from 6-wk pre-breeding up to 2-wk post-breeding.

2 ADG, average daily gain; TMR, total mixed ration.

3 CON (*n *= 30) and FLAX (*n *= 26). Average SEM for treatment × week interaction.

Abbreviations: BW, body weight; d, day; FLAX, flaxseed oil-based supplement; kg, kilogram; min, minute.

**Figure 2.**
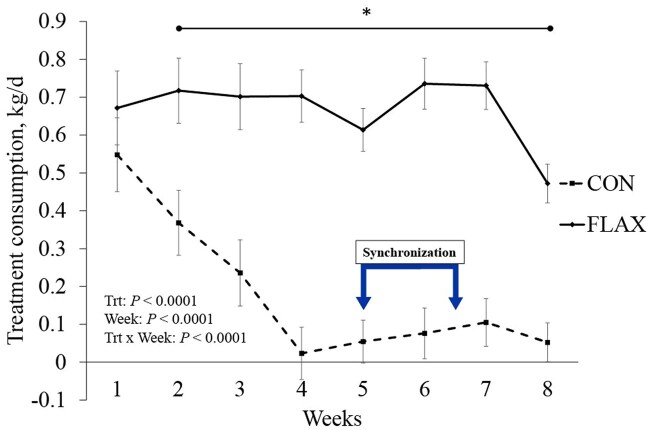
Treatment intake of beef heifers (CON, loose mineral supplement [*n *= 30] vs. FLAX, flaxseed oil-based supplement delivered via low moisture block [*n *= 26]). Treatments were applied from 6-wk pre-breeding up to 2-wk post-breeding, thus totaling 8 wk. Measurements were compiled by kg/d for each heifer and averaged for each week. Arrow segment represents weeks where fixed-time artificial insemination protocol was performed. Asterisk represents significant differences between treatments (*P* ≤ 0.05). Abbreviations: d, day; FLAX, flaxseed oil-based supplement; kg, kilogram; Trt, treatment.

The average supplement intake (Figure 2) across the trial was greater in FLAX compared with CON, showing a treatment × week interaction (*P *< 0.01), where the CON group exhibited a sharp decline in treatment consumption over the trial period after week 2. In contrast, the FLAX group had consistently greater treatment consumption across the experimental period from the first week. Similarly, a treatment × week interaction (*P *< 0.01) for treatment intake duration (Table 4) showed a drastically greater duration in FLAX heifers for weeks 2 through 8.

**Figure 3.**
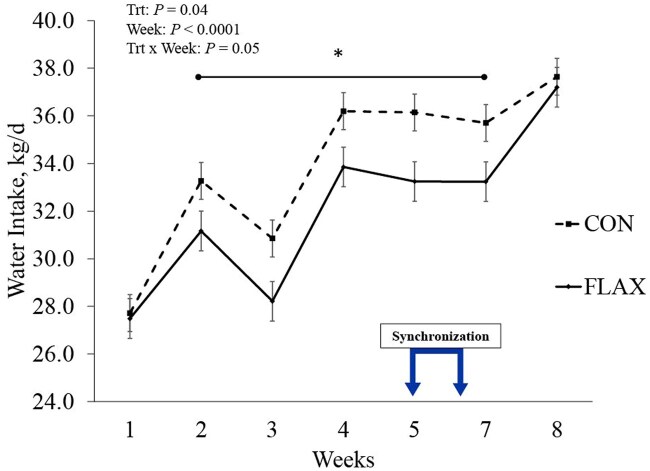
Water intake of beef heifers (CON, loose mineral supplement [*n *= 30] vs. FLAX delivered via low moisture block [*n *= 26]) on. Treatments were applied from 6-wk pre-breeding up to 2-wk post-breeding, thus totaling 8 wk. Measurements were compiled by kg/d for each heifer and averaged for each week. The “Synchronization” segment represents weeks where fixed-time artificial insemination protocol was performed. Asterisk represents significant differences between treatments (*P* ≤ 0.05). Abbreviations: d, day; FLAX, flaxseed oil-based supplement; kg, kilogram; Trt, treatment.

Water intake (Figure 3) was affected by treatment × week interaction (*P *≤ 0.05). Overall, water intake was lower for FLAX than CON heifers in weeks 1, 2, 4, 5, and 7.

**Figure 4.**
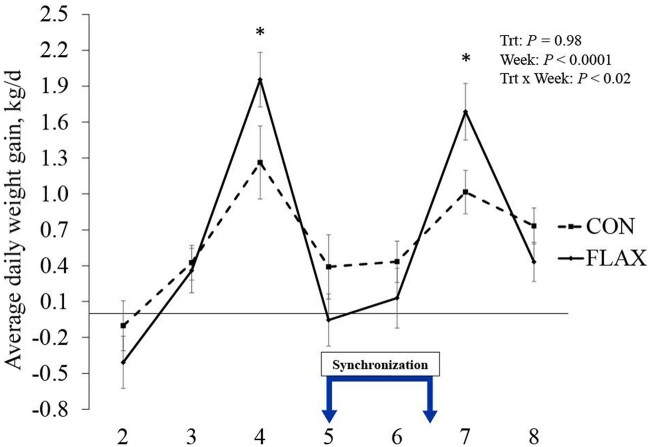
Average daily weight gain of beef heifers (CON, loose mineral supplement [*n *= 30] vs. FLAX delivered via low moisture block [*n *= 26]). Treatments were applied from 6-wk pre-breeding up to 2-wk post-breeding, thus totaling 8 wk. Measurements were compiled by kg/d for each heifer and averaged for each week. The “Synchronization” segment represents weeks where fixed-time artificial insemination protocol was performed. Asterisk represents significant differences between treatments (*P* ≤ 0.05). Abbreviations: d, day; FLAX, flaxseed oil-based supplement; kg, kilogram; Trt, treatment.

ADG (Figure 4) exhibited a treatment week interaction (*P *= 0.03), being greater in FLAX heifers during weeks 4 and 7 but lower in week 5. However, ADG did not differ between treatment groups overall (*P *= 0.97).

### Puberty attainment

**Figure 5.**
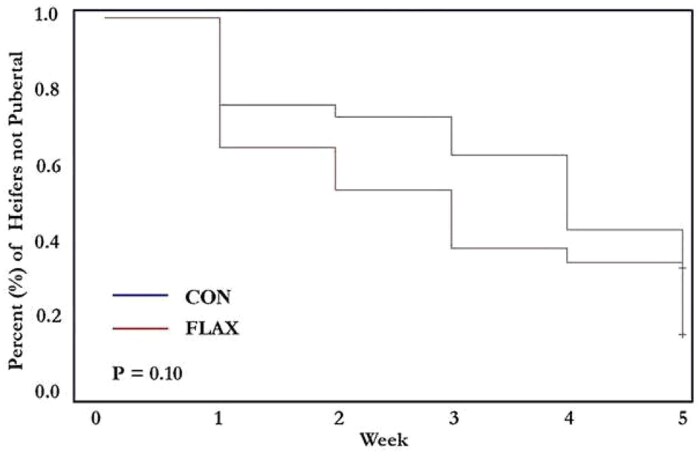
Proportion of pubertal beef heifers (CON, loose mineral supplement [*n *= 30] vs. FLAX, flaxseed oil-based supplement delivered via low moisture block [*n *= 26]) by the time of induction. On the *x*-axis are the weeks and on the *y*-axis is the probability of a heifer NOT attaining puberty by that week.

The cumulative percentage of heifers that had a CL detected on ultrasound by week 5 was similar between CON and FLAX (16/30, 53.3% for CON vs. 18/26, 69.2% for FLAX; *P *= 0.24). However, the FLAX heifers tended (*P *= 0.10, Figure 5) to achieve puberty faster than the CON group by the time of puberty induction (first day of FTAI protocol).

### Largest follicle diameter, plasma P4, E2 and pregnancy rates

The effects of flaxseed oil supplementation on LFD, P4, E2, and pregnancy rates are summarized in Table 5. There was no interaction between treatment and day for LFD between groups (*P *= 0.54). While there was a difference in LFD across days (*P *< 0.01), no differences in LFD were observed between treatments for D0 (*P *= 0.30), D7 (*P *= 0.74), or D9 (*P *= 0.20).

**Table 5.** Supplementation strategy (CON, loose mineral supplement vs. FLAX via low moisture block) impact on largest follicle diameter, concentrations of progesterone, and estradiol, and pregnancy rates of beef heifers

	Treatment^1^				*P*-value	
Day of synchronization^2^	FLAX	CON	SEM^3^	Treatment	Day	Treatment × day
LFD (mm)						
D0	12.15	13.37	0.81	0.30		
D7	12.88	13.17	0.82	0.74	<0.01	0.54
D9	15.35	16.26	0.51	0.20		
P4 (ng/mL)						
D0	2.21	1.24	0.47	0.15		
D7	3.29	2.39	0.56	0.25	–	–
D9	0.46	0.77	0.12	0.09		
E2 (pg/mL)						
D7	4.06	3.56	0.27	0.20	–	–
D9	4.22	4.35	0.34	0.81		
P/AI (%, N/N*/*)						
∼D32 after AI	69.2 18/26	60.0 18/30	0.09	0.48	–	–
∼D60 after AI	88.5 23/26	86.7 26/30	0.06	0.84	–	–

1 Treatments were applied from 6-wk pre-breeding up to 2-wk post-breeding.

2 Days 0, 7, and 9 of synchronization and pregnancy rates for days ∼32 and ∼60 after artificial insemination.

3 CON (*n *= 30) and FLAX (*n *= 26). Average SEM for the treatment.

Abbreviations: d, day; E2 = estradiol; FLAX, flaxseed oil-based supplement; LFD, largest follicle diameter; mL, milliliter; mm, millimeter; N, nanogram; P/AI, pregnancies per artificial insemination; pg, picogram; P4 = progesterone.

Circulating E2 concentrations were not affected by treatment, with no significant difference on D7 (*P *= 0.20; Table 4) or D9 (*P *= 0.81) between FLAX and CON heifers.

In addition, circulating P4 concentration (Table 5) was not affected by treatment on D0 (*P *= 0.15) or D7 (*P *= 0.25). However, on D9, the day of AI, there was a tendency for P4 concentration to be lower in FLAX heifers than CON (*P *= 0.09). The P/AI (Table 5) was also not significantly affected by supplementation at D32 (*P *= 0.48) or D60 (*P *= 0.84) for FLAX and CON groups, respectively.

### Lipid profile

The overall effects of flaxseed oil-based supplementation on plasma FA concentrations are presented in Table 6. There were significant treatment × week interactions for multiple FAs. For ALA, concentrations increased (*P *= 0.05) in FLAX heifers, while CON showed no significant change. Linoleic acid (LA) concentrations in FLAX heifers increased (*P *= 0.01), while CON showed no change. Total PUFA concentrations demonstrated a similar trend, with FLAX concentrations increasing (*P *= 0.05) compared with a smaller increase in CON. AA tended (*P *= 0.09) to exhibit a treatment × week interaction, with FLAX concentrations increasing from week 1 to week 8, while CON showed no significant change.

**Table 6.** Supplementation strategy (CON, loose mineral supplement vs. FLAX delivered via low moisture block) impact on concentrations of fatty acids in the plasma of beef heifers

		Weeks			*P*-values		
Fatty Acid (µg/mL)	Treatment^1^	1	8	SEM^2^	Treatment	Week	Treatment × week
α-Linolenic (C18:3)	FLAX	15.48^a^	16.73^b^	0.20	0.03	0.0002	0.05
	CON	15.44^a^	15.86^a^				
Eicosapentaenoic (C20:5)	FLAX	3.61	4.06	0.19	0.07	0.22	0.38
	CON	3.47	3.54				
Docosahexaenoic (C22:6)	FLAX	3.27	5.21	2.39	0.67	0.22	0.23
	CON	5.98	5.19				
Linoleic (C18:2)	FLAX	39.52^a^	60.11^b^	3.92	0.02	0.002	0.01
	CON	38.13^a^	40.35^a^				
Arachidonic (C20:4)	FLAX	2.46	3.00	0.23	0.10	0.05	0.09
	CON	2.27	2.32				
Total PUFA	FLAX	103.79^a^	130.5^b^	5.76	0.18	0.002	0.04
	CON	105.76^a^	111.19^a^				

1 Treatments were applied from 6-wk pre-breeding up to 2-wk post-breeding. For fatty acid analysis, blood was collected at the beginning (week 1) and end of the trial (week 8).

2 CON (*n *= 30) and FLAX (*n *= 26). Average SEM for treatment × week interaction.

a,b within a row without a common superscript denotes differences between treatments (*P *≤ 0.05). Abbreviations: FLAX, flaxseed oil-based supplement; PUFA, polyunsaturated fatty acids.

There was a tendency for greater concentrations of eicosapentaenoic acid (EPA; *P *= 0.07). Docosahexaenoic acid was not significantly affected by treatment (*P *= 0.69) or time (*P *= 0.67).

## Discussion

This study aimed to investigate the effects of a flaxseed oil-based supplementation on reproductive parameters, feeding behavior, and lipid profile in beef heifers. The results demonstrate that the FLAX significantly altered the lipid profile and feeding behavior. In addition, supplementation demonstrated the tendency to alter P4 concentrations along with puberty attainment. These results suggest that supplementation of n-3 PUFA presented as a flaxseed oil-based block has the potential to impact heifer production, but more studies are needed to narrow down these effects.

There is a wide variation in supplementation strategies among producers (Kunkle et al., 2000). Providing loose mineral supplementation is one of the most common ways to overcome potential mineral deficiencies. However, growing heifers (especially first-calf heifers) have greater nutrient demands (maintenance, growth, gestation, lactation) than mature cows, so providing a combination of protein/energy + mineral/vitamin supplements would be beneficial for this category of cattle (Larson et al., 2016).

Heifers consuming FLAX exhibited a greater intake of treatments for both kg/d consumed, and min/d spent at the feeder compared to CON heifers. This finding could be attributed to the novelty of the LMB and possibly a form of enrichment for confinement cattle (Pelley et al., 1995). This would agree with findings of more consumption of supplement by cattle housed in dry lot pens offered LMB compared to hand-fed granular concentrate (Brandão et al., 2020). The TMR may have also provided enough sufficient nutritional value to reduce CON supplement intake. Poppi and McLennan (1995) extensively reviewed nutrient balance and how it can be affected by metabolic regulation. This also highlights that cattle on pasture and forage-based diets may benefit more from these free-choice supplementation strategies than those fed a TMR. While the novelty of an LMB supplement may encourage initial intake, there are factors such as block composition and competition between animals that contribute to its variability (Moriel et al., 2019). Variability in intake was reported previously in a study comparing a loose mineral supplement mix and a molasses-based block supplement over 10 wk in beef heifers (Dixon et al., 2003). While the study showed high variability (55–95% variation) in both groups, a tendency was observed for a greater percentage of heifers in the block supplement group to not consume the product at all, contrasting with the findings of the present study. This variability could be attributed to the unfamiliarity of this presentation, which some animals need more time to adjust to. This novelty may also cause competition, especially in larger herds (Chapple and Lynch, 1986).

The impact of flaxseed-oil based supplementation was further exhibited through a tendency suggesting that the LMB may affect TMR intake differently from the CON group over time, which is consistent with findings from Sun et al. (2023) in Holstein cows fed extruded flaxseed, extruded soybean meal, or a saturated fatty acid (SFA) control diet. The flaxseed-supplemented group tended to show greater intake of TMR than the other groups. This could be attributed to the many factors affecting TMR intake, including the type and palatability of supplemented oilseeds, the forage-to-concentrate ratio, and energy balance status (Zachut et al., 2010). While FLAX heifers tended to consume more TMR than CON heifers, there was no difference in ADG or final BW. One study found similar results in Charolais heifers supplemented with partially rumen-protected long-chain PUFA as a source of n-3 or palmitic acid as the control diet (Hennessy et al., 2021). Diet did not influence TMR intake, average daily gain (ADG), or BCS for heifers in this study. These results on performance are similar to the present study, suggesting that the most advantageous effects of n-3 PUFA supplementation may rely on reproductive performance.

Additionally, FLAX heifers consumed less water than CON. The addition of salt to the diet has been shown to increase water intake in cattle (Riggs et al., 1953). Sodium is a regulator of water and electrolyte balance, which may explain these findings (Berger, 2006). This may also be a factor causing an increase in water consumption for the CON group, as the FLAX supplement contained no added sodium.

Another notable difference in supplement composition between the FLAX and CON groups is the added crude protein (CP) of the flaxseed oil LMB. CP can cause detrimental effects when fed in excess, as well as when in restriction in relation to the requirements. Intake of CP above requirements has been shown to increase serum albumin and total serum protein, which negatively affects days open, services per conception, and days to first estrus (Jordan and Swanson, 1979). Further, Holstein cows and heifers fed a high-protein diet (19% CP) had higher plasma urea concentrations and decreased first-service conception rates compared to those fed a moderate-protein diet (16% CP) with no differences seen in energy balance, dry matter intake (DMI), or milk production (Canfield et al., 1990). Protein deficiency results in infertility, reduced fetal weight, and decreased milk production, with resultant decreases in the weaning weights of calves and decreased DMI (Maas, 1987). Concerning the present study, the TMR was formulated to meet CP requirements by itself for both groups; hence, the FLAX group exceeded the CP total. However, the lack of detrimental results in that group provides evidence that flaxseed oil is the ingredient that impacted the heifers.

Supporting our initial hypothesis, the results showed a distinct difference in the lipid profile of heifers fed the FLAX compared to the control. The significant increase in ALA and LA by week 8 demonstrated the effectiveness of the supplement in being metabolized to successfully modify the circulating lipid profile. The n-6 FA precursor LA is an important substrate for prostaglandin synthesis (Carroll et al., 1992). Specifically, AA derived from the desaturation and elongation of linoleic acid is a precursor for PGF2α, which serves an important role in CL regression and initiation of a new estrous cycle (Mattos, 2000; Funston, 2004). The FLAX group also tended to have greater concentrations of EPA and AA. These findings were expected and are consistent with previous literature using n-3 PUFA supplementation (Childs et al., 2008; Maranesi et al., 2018). Alterations in specific FA, such as EPA and AA, may have implications for the pro-inflammatory and anti-inflammatory processes involved in overall reproductive mechanisms (Mattos, 2000). In addition, Hinckley et al. (1996) reported that the presence of n-3 FA attenuates the rate of n-6 eicosanoid production, which could explain the increased levels of both n-3 and n-6 FA and associated derivatives in this study.

Derivatives and byproducts associated with FA supplementation may also affect puberty attainment. Supplementation with PUFA has been shown to increase cholesterol in cattle (Jolazadeh et al., 2019), which is an important substrate in the process of steroidogenesis. This may aid in accelerating puberty attainment. The FLAX group heifers tended to attain puberty earlier than the non-supplemented group (CON), which is consistent with prior findings utilizing various sources of n-3 PUFA, specifically rice bran and palm oil (Lammoglia et al., 2000). Heifers supplemented with n-3 PUFA in the form of palm oil reached puberty 4 months earlier than their non-supplemented counterparts (Wafa et al., 2022). Several other studies found no significant effect of fat supplementation from cottonseed, pelleted soyhulls, or whole soybeans on puberty attainment (Howlett et al., 2003). Whole sunflower seed, however, was shown to delay puberty in crossbred beef heifers (Garcia et al., 2003). Sunflower seed is a source of n-6 FA containing high levels of LA. It was hypothesized that this may cause a delay in puberty attainment as conjugated linoleic acid has been correlated with lower adiposity, which is essential for puberty attainment (Gillis et al., 2004). Those studies associated with our findings emphasize that the potential to improve reproductive performance when using lipid supplementation is directly associated with the source and presentation of the FAs.

In terms of the endocrine environment, one study investigating the effects of n-3 PUFA supplementation on hormone production utilized fish oil at three different levels in heifers with a non-supplemented control group (Childs et al., 2008b). Similar to the present study, there was no effect of diet on P4 or E2 concentrations in any treatment group. The lack of a diet effect on E2 could be attributed to n-3 FA-mediated reduction in the activity of cholesterol ester transfer protein, causing less cholesterol transfer from high-density lipoprotein (HDL) to very-low-density lipoprotein (VLDL) and low-density lipoprotein (LDL) (Nestel, 2000). This effect, coupled with n-3 PUFA’s ability to lower triglyceride concentrations, has been speculated to increase circulating cholesterol. This may result in greater P4, although this was not the outcome of the present study.

Overall, this study shows that n-3 supplementation via flaxseed oil does not seem to impact the LFD or P4 on D0 and D7 of an FTAI protocol, or conception rates in beef heifers. However, it can influence certain important aspects of reproductive efficiency, such as puberty attainment, and affect the endocrine environment by altering the circulating lipid profile. The tendency for earlier puberty in FLAX heifers suggests that n-3 FAs may play a role in accelerating reproductive maturity, which may aid in managing reproductive efficiency in beef production systems (Perry and Cushman, 2013). Additionally, the observed changes in the lipid profile, particularly the increase or tendency to increase FAs needed for prostaglandin synthesis, suggest potential health benefits or improvements in overall physiological function by mediating pro- and anti-inflammatory actions (Abayasekara and Wathes, 1999). However, further research is necessary to elucidate the effects of such supplementation. The present study shows promising results regarding increased treatment consumption, decreased water intake, plasma lipid alterations, accelerated puberty attainment, and P4 impacted by supplementation of flaxseed oil. However, there is a need to explore the direct mechanisms through which n-3 PUFA in flaxseed oil influence these metabolic and reproductive processes at the cellular level.

## Abbreviations:

AA: arachidonic acid
ADG: average daily gain
AI: artificial insemination
AIC: Akaike information criterion
ALA: α-linolenic acid
BW: body weight
CCERF: Cow–Calf Education and Research Facility
CIDR: controlled internal drug release
CL: corpus luteum
E2: estradiol
EPA: eicosapentaenoic acid
FA: fatty acid
FTAI: fixed-time artificial insemination
GnRH: gonadotropin releasing hormone
LFD: largest follicle diameter
LA: linoleic acid
LMB: low-moisture block
n-3: omega-3
n-6: omega-6
P4: progesterone
P/AI: pregnancy per artificial insemination
PGF: prostaglandin F2α
PUFA: polyunsaturated fatty acid
